# Change in the Foveal Avascular Zone and Macular Capillary Network Density after Hyperbaric Oxygen Therapy in Healthy Retina

**DOI:** 10.18502/jovr.v16i3.9436

**Published:** 2021-07-29

**Authors:** Sadık Görkem Çevik, Bekir Selim Bağlı

**Affiliations:** ^1^Department of Ophthalmology, Yuksek Ihtisas Education and Training Hospital, Bursa, Turkey; ^2^Department of Underwater and Hyperbaric Medicine, Yuksek Ihtisas Education and Training Hospital, Bursa, Turkey

**Keywords:** Hyperbaric Oxygen, OCT-A, Retinal Ischemic Disease

## Abstract

**Purpose:**

This study aimed to evaluate responses in retinal tissue by swept source OCT angiography (OCT-A) to hyperoxia after hyperbaric oxygen (HBO2) therapy.

**Methods:**

The study was conducted in volunteers who received HBO2 treatment but did not have any eye disease. Patients underwent detailed eye examinations including dilated fundus examination, visual acuity, and refraction before being admitted for HBO2 therapy. Measurements were made before and immediately after HBO2 therapy. Enface images of the retinal vasculature were obtained from the superficial and deep retinal plexus (SP/DP). Quantitative analysis of the vessel density (VD) and foveal avascular zone (FAZ) area was performed.

**Results:**

In total, 31 patients (15 female) with healthy retina were included in the study. The mean age was 42.8 years. The mean SP vascular density measurements before HBO2 therapy for the right and left eyes were 15.18 ± 1.2 mm ${}^{-1}$ and 15.01 ± 1.3 mm ${}^{-1}$ , respectively; the measurements after HBO2 therapy for the right and left eyes were 14.34 ± 1.4 mm ${}^{-1}$ and 14.48 ± 1.19 mm ${}^{-1}$ . The mean DP vascular density measurements before HBO2 therapy for the right and left eyes were 16.03 ± 1.69 mm ${}^{-1}$ and 16.1 ± 1.45 mm ${}^{-1}$ , respectively; the measurements after HBO2 therapy for the right and left eyes were 15.02 ± 1.65 mm ${}^{-1}$ and 15.12 ± 2.16 mm ${}^{-1}$ , respectively. Reduction of mean VD in superficial and deep plexus after HBO2 was statistically significant (*P* = 0.001 and *P* = 0.000, respectively). Changes in mean FAZ area before and after HBO2 therapy were not statistically significant (*P* = 0.719).

**Conclusion:**

The healthy retina responds to oxygen supersaturation with HBO2 therapy by eventually decreasing vascular density in all layers. These findings may be important for further studies especially related to retina and choroidal oxygenation.

##  INTRODUCTION

Hyperbaric oxygen (HBO2) therapy is a medical treatment method that aims to increase the level of dissolved oxygen in tissues. Patients intermittently breathe near 100% oxygen while inside a hyperbaric chamber that is pressurized to greater than sea level pressure (1 atmosphere absolute [ATA]) and high tissue oxygen level remains high for up to 4 hr after the therapy.^[1]^ Retina blood flow is provided by two primary sources: the retinal artery and the choroid. The retina has an internal autoregulatory system that preserves blood flow and stable oxygenation by altering it in response to unexpected conditions.^[2]^ This regulatory system changes blood flow and vascular density for unexpected conditions and may not work correctly under some conditions, such as diabetic retinopathy or age-related macular degeneration.^[3,4,5,6,7,8,9]^

Macular microvasculature is a complex system consisting of three capillary plexuses for the blood supply of the inner retina: the superficial retinal plexus (SP) located in the retinal nerve fiber layer and the two-plexus located at the inner and outer border of the inner nuclear layer (INL), which constitute the deep retinal plexus (DP).^[10]^ Noninvasive retinal blood-flow imaging techniques, such as scanning laser Doppler and Doppler OCT, have previously been described.^[11,12,13,14]^ OCT angiography (OCT-A) is a novel, noninvasive technique for retinal vasculature imaging that allows segmentation and quantification of the retinal microvasculature.^[15]^

The present study aimed to evaluate changes in foveal avascular zone (FAZ) area and perifoveal capillary network density in SP and DP, with the aid of swept source OCT-A in patients undergoing HBO2 therapy.

##  METHODS

This prospective, interventional study was handled between April 2017 and October 2017 after the ethics committee of hospital approved the protocol. The study adhered to the Declaration of Helsinki, and patients were informed about the treatment options and possible complications. The preprocedural written informed consent was obtained from patients. The study was carried on in volunteers who did not have any known eye disease and who received HBO2 treatment for other reasons. Volunteer patients underwent detailed eye examinations that included dilated fundus examination and visual acuity and refraction before they were admitted for HBO2 therapy. Exclusion criteria were: age < 18 years; any ocular diseases; inability to maintain stable fixation for scanning; visual acuity worse than 20/40; refractive errors >- 4.00 or +2.00 diopters; media opacity; history of ocular surgery; and systemic diseases that may affect microc0irculation (e.g., diabetes mellitus, hypertension).

**Figure 1:**
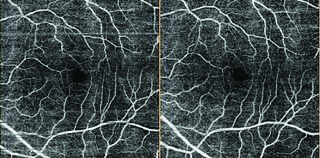
Swept source OCT-A image before (left) and after (right) HBO2 treatment (superficial plexus).

Measurements were made using the DRI OCT Triton (Topcon Co- Tokyo-Japan) system before and immediately (approximately 1 min) after the fifth session of HBO2 therapy (every day one session). FAZ, superficial vascular plexus (SP) [Figure 1] and deep vascular [Figure 3] plexus (DP) density measurements were made.

**Figure 2:**
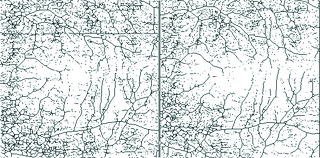
Skeletonized vessel maps of the superficial plexus image before (left) and after (right) HBO2 treatment.

**Figure 3:**
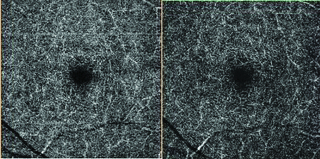
Swept source OCT-A image before (left) and after (right) HBO2 treatment (deep plexus).

**Figure 4:**
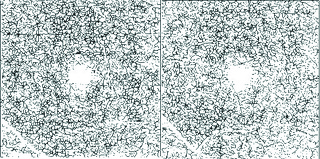
Skeletonized vessel maps of the deep plexus image before (left) and after (right) HBO2 treatment.

The DRI OCT Triton system uses a swept-source laser with a center wavelength of 1050 nm and a scan speed of 100,000 A-scans per second. OCT-A is based on the Topcon OCT Angiography ratio analysis (OCTARA) algorithm. This system distinguishes mobile blood flow from tissue using motion contrast measure. An active eye tracker was used to decrease motion artifacts during OCT-A imaging. Each 3 × 3 mm volume scan contained 320 × 320 pixels. In the present study, we applied automated layer segmentation to SP (from 2.6 μm below the internal limiting membrane to 15.6 μm below the junction between the inner plexiform and inner nuclear layers [IPL/INL]) and DP (from 15.6 μm below of IPL/INL to 70.2 μm below the IPL/INL). All measurements were obtained between 9:00 AM and 12:00 AM to avoid the effects of diurnal variations and blood pressure and pulse were recorded. Poor-quality OCT-A due to blinking or fixation loss were excluded from the study. The qualitative examination of the OCT-A of the SP and DP were then independently performed by two masked examiners (Dr Akdogan and Dr Tok).

Quantitative analysis of 3 × 3 mm OCT angiograms, including FAZ area and capillary vessel density (CVD; i.e., SP–DP) measurement, was performed using ImageJ software (developed by Wayne Rasband, National Institutes of Health, Bethesda, MD; available at http://rsb.info.nih.gov/ij/index.html). The original OCTA images of the SP and the DP were binarized to convert them from grayscale into black and white images using the ImageJ software, and the FAZ area was manually marked and measured in original images of SP.

To measure the CVD, the binarized OCTA images were skeletonized, showing the blood vessels as a 1-pixel-wide line, and ImageJ was used to count the number of black pixels and total pixels [Figures 2 and 4]. CVD was then calculated as ([pixels of vessels,3/256])/(area in squared millimeter) in mm ${}^{-1}$ and this method was described by Khairallah et al previously.^[27]^ Cases with projection artifact were excluded from the analysis of vessel density. HBO2 therapy was administered in multiple chambers at 2.4 ATA. Each session of HBO2 therapy lasted about 120 min composed of three 25-min oxygen periods during which patients breathed 100% oxygen at 2.4 ATA, separated by 5-min air brakes, with compression and decompression during the remaining time.

Data analysis was performed using SPSS Statistics for Windows, version 22.0 (SPSS Inc., Chicago, IL, United States). All differences associated with a chance probability of 0.05 or less were considered statistically significant. Statistical significance between before and after HBO2 therapy was tested with dependent samples *t*-test.

##  RESULTS

This study included 31 patients (15 female, 48%) with healthy retinas. The mean age was 42.8 ± 15.3 (19–73) years. Indications of HBO2 therapy were femur bone avascular necrosis (15 patients, 48%), chronic osteomyelitis (8 patients, 25%), idiopathic sudden sensorineural hearing loss (6 patients, 19%), malign otitis externa (1 patient, 3%), crush injury (1 patient, 3%), as summarized in Table 1.

**Table 1:** Indications of HBO2 therapy for patients


**Indications**	**Number of patients**
Femur bone avascular necrosis	15 (48 %)
Chronic osteomyelitis	8 (25 %)
Idiopathic sudden sensorineural hearing loss	6 (19 %)
Severe otitis externa	1 (3 %)
Crush injury	1 (3 %)
Total	31

**Table 2:** Measurement data before and after HBO2 therapy


	**Before HBO2 therapy**	**After HBO2 therapy**	***P*** **-value**
Mean right eye superficial capillary density (mm ${}^{-1}$ )	15.18 ± 1.2	14.34 ± 1.4	0.001
Mean right eye deep capillary density (mm ${}^{-1}$ )	16.03 ± 1.69	15.02 ± 1.65	0.000
Mean left eye superficial capillary density (mm ${}^{-1}$ )	15.01 ± 1.3	14.48 ± 1.19	0.001
Mean left eye deep capillary density (mm ${}^{-1}$ )	16.1 ± 1.45	15.12 ± 2.16	0.000
Mean right eye FAZ (mm ${}^{2}$ )	0.363	0.372	0.374
Mean left eye FAZ (mm ${}^{2}$ )	0.354	0.357	0.719

The mean SP vascular density measurements before HBO2 therapy for the right and left eyes were 15.18 ± 1.2 mm ${}^{-1}$ (min 13.1 mm ${}^{-1}$ – max 17.1 mm ${}^{-1}$ ) and 15.01 ± 1.3 mm ${}^{-1}$ (min 13.3 mm ${}^{-1}$ – max 17.2 mm ${}^{-1}$ ) respectively; the measurements after HBO2 therapy for the right and left eyes were 14.34 ± 1.4 mm ${}^{-1}$ (min 12.1 mm ${}^{-1}$ – max 16.2 mm ${}^{-1}$ ) and 14.48 ± 1.19 mm ${}^{-1}$ , (min 12.3 mm ${}^{-1}$ – max 16.2 mm ${}^{-1}$ ) respectively. These changes were statistically significant (*P* = 0.001 and *P* = 0.001, respectively).

The mean DP vascular density measurements before HBO2 therapy for the right and left eyes were 16.03 ± 1.69 mm ${}^{-1}$ (min 13.82 mm ${}^{-1}$ – max 18.12 mm ${}^{-1}$ ) and 16.1 ± 1.45 mm ${}^{-1}$ (min 13.82 mm ${}^{-1}$ – max 18.03 mm ${}^{-1}$ ), respectively; the measurements after HBO2 therapy for the right and left eyes were 15.02 ± 1.65 mm ${}^{-1}$ (min 13.23 mm ${}^{-1}$ – max 17.05 mm ${}^{-1}$ ) and 15.12 ± 2.16 mm ${}^{-1}$ (min 12.10 mm ${}^{-1}$ – max 18.23 mm ${}^{-1}$ ), respectively. These changes were statistically significant (*P* < 0.000 and *P* < 0.000, respectively). (Measurement data before and after HBO2 therapy are shown in Table 2.)

The mean FAZ area for the right eye was 0.363 ± 0.15 (0.20–0.62) mm ${}^{2}$ before HBO2 therapy and 0.372 ± 0.34 (0.21–0.49) mm ${}^{2}$ after the therapy. The mean FAZ area for the left eye was 0.354 ± 0.32 (0.28–0.47) mm ${}^{2}$ before HBO2 therapy and 0.357 mm ${}^{2}$ ± 0.22 (0.23–0.46) after the therapy. These changes were not statistically significant (*P* = 0.374, *P* = 0.719).

##  DISCUSSION

In the present study, we aimed to investigate the effect of five sessions HBO2 therapy on the retina. We found significant decreases in SP and DP capillary network density in the macular region. However, there was no effect on FAZ measurements.

The retina has unique dual circulation and complicated autoregulatory systems. The outer retina and FAZ are nourished with diffusion from the choroidal circulation (CC).^[20,26]^ Responses to hypoxia in retinal artery circulation (RC) and CC are different.^[18]^ Yi et al reported that CC responds poorly in hypoxia conditions.^[19]^

Linsenmeier and Braun found that the inner retina was well-preserved under hypoxic condition by the help of increased RC during hypoxia, while blood flow in CC remained unchanged in cats' retina.^[20]^ Retinal response to hyperoxia is different between mammalian species. The rat, rabbit, and guinea pig that are commonly demanded mammalian in ophthalmic research and they have different responses to systemic hyperoxia.^[3]^ Hyperoxia changes total human retinal blood flow, as measured by other measurement methods before.^[13,14,15,16]^ The autoregulatory response protects retinal tissue from the adverse effects of hypoxia and hyperoxia. Hagag et al found that hyperoxia caused a significant decrease in the flow index (11%) and the VD of the deep capillary plexus (DP) of the retina (7.8%). However, decreases in the vessel densities of the superficial and intermediate plexuses were not statistically significant. In Hagag et al's study, oxygen was supplied with a face mask for individuals; the mask flow rate was 15 l/min.^[17]^ Hagag et al hypothesized that the response of DP in different oxygen concentrations could be significant and essential because CC did not respond like DP and the blood flow of the SP also decreased in over-oxygenation but supplying oxygen with a face mask may not be enough to produce a statistically significant decrease. Contrary to Hagag et al's study, reduction in both SP and DP VD was statistically significant in our study. We think that this may be the result of a high amount of dissolved oxygen in the plasma that was supplied by HBO2. HBO2 therapy increased dissolved oxygen levels in all retinal layers, including the superficial retinal layers. During HBO2, the oxygen concentration in the avascular outer retina increases due to higher supply from the CC, which maintains constant flow at high O ${}_{2}$ level. To preserve steady oxygen flux in the outer retina, the DP must compensate with greater vasoconstriction.^[25,28,29,30]^

In the present study, the changes in FAZ before and after HBO2 therapy were not statistically significant. FAZ area changes can be seen in retinal disease and five HBO2 sessions may be short to describe certain effect of HBO2 on FAZ.^[21,22,23,24]^

The limitations of our study are: small sample size, limited number of HBO2 sessions, measurement of vascular plexus density by an external software, and assessment of only 3 × 3 mm macular area.

In summary, swept source OCT-A evaluate retinal vascular plexus and FAZ and we reported a significant decrease of foveal capillary network density in SP and DP with HBO2 therapy. This finding gives us new evidence regarding the interaction between choroidal and retinal circulation and offers us new perspective about retinal oxygenation with HBO2 treatment.

##  Financial Support and Sponsorship

Nil.

##  Conflicts of Interest

There is no conflict of interest.
